# Lipid droplets in felid kidneys: prevalence and composition by lipidomics

**DOI:** 10.3389/fvets.2026.1711591

**Published:** 2026-02-23

**Authors:** Rebecca A. Brociek, Rebecca Alborough, Anna M. Kotowska, Ana Ferreira, Sandra Martinez-Jarquin, Malgorzata Walczak, Vincenzo Di Bari, Frederic Beaudoin, David S. Gardner

**Affiliations:** 1 School of Veterinary Medicine and Science, University of Nottingham, Nottingham, United Kingdom; 2 School of Pharmacy, University of Nottingham, Nottingham, United Kingdom; 3 School of Biosciences, University of Nottingham, Nottingham, United Kingdom; 4 Rothamsted Research, Harpenden, United Kingdom

**Keywords:** cat, felids, lipid droplet, lipidomics, nutrition

## Abstract

**Introduction:**

An accepted and common phenotypic curiosity of Felidae is the presence of intracytoplasmic lipid droplets in renal proximal tubule epithelial cells (RPTEC), also frequently observed in urine (lipuria). Both outcomes are currently considered, and taught, as incidental – without obvious pathophysiological consequence. This contrasts markedly with clinical (human) medicine, where lipid vacuoles in RPTEC are usually associated with metabolic or chronic disease, such as chronic kidney disease (CKD). Despite domestic felids having a high incidence of CKD with advancing age, no study has fully characterized feline RPTEC lipid droplets in the context of CKD.

**Methods:**

In this study, we first characterized the incidence of RPTEC lipid in domestic cats (with/without CKD or chronic interstitial nephritis) versus domestic dogs and Scottish Wildcats, across a wide age range, using a number of lipidomic approaches (chromatography, fatty acid characterization, mass spectrometry).

**Results:**

Felids (domestic and wildcat) consistently had greater renal lipid content than dogs at all ages studied. Intracytoplasmic lipid extraction revealed a panoply of novel lipids found only in domestic cats: lipids were primarily modified (i.e., less polar) ether-soluble triacylglycerols, including mono-alkyl-diacylglycerols (MADAGs) and other likely, branched-chain fatty acids.

**Discussion:**

We suggest that the common presence of such rare lipid species in tubular lipid droplets in domestic cats reflects an aspect of felid biology that parallels age-related disease prevalence, in particular, being associated with the aetiopathogenesis of chronic renal interstitial nephritis (CIN) – a hallmark of CKD in felids.

## Introduction

In humans and most other mammalian species, renal intracellular lipid deposition is an adverse finding associated with metabolic syndrome and/or obesity (1). Ectopic lipid (i.e., lipid not esterified and stored in adipose tissue) can provoke and initiate renal cell damage through lipotoxic processes, which may eventually result in chronic kidney disease (CKD) (2–4). However, in domestic cats (DC), the presence of a large quantity of intracytoplasmic lipid droplets within the renal tubular epithelial cells, and in urine – lipuria – is accepted as an ‘incidental’ finding; that is, an observation with no known adverse outcome (5). Such early reports in the field of veterinary medicine are likely based on the fact that the droplets are so prevalent in cats and do not appear to be associated with body weight or other co-morbidities (6). Thus, ‘incidental findings’ have become lingua franca in small animal veterinary medicine with regard to the presence of lipid in felid kidneys.

In the domestic cat, renal lipid is mostly found in the proximal tubule epithelial cells (RPTEC) of the renal cortex (7, 8), although some deposits have been noted in the interstitium alongside macrophages (9). RPTEC lipid droplets or ‘lipid vacuoles’ have also been observed in wild or captive wild cats such as lions and tigers, although the incidence is relatively low (10, 11). Nevertheless, lipuria remains a relatively common clinical pathological finding in wild cats (10, 12). Rarely are such phenotypes observed in domestic dogs (which share the similar domestic environment as DCs) or other mammals (7). Advanced age is one factor known to increase the presence of renal lipids in both cats and in dogs (13). Indeed, Quimby et al. (14) reported that 79% senior cats have renal lipid accumulation. Remarkably, the same study reported 25% cats aged between 1 and 5 years also presented with renal lipid (14), and at least some lipid droplets have been noted in kittens (6).

Presently, no study using advanced techniques to characterize lipids has determined the composition of such intracytoplasmic RPTEC lipids in felids. An early study proposed that the lipid droplets were mainly triacylglycerides, phospholipids, and cholesterol (15). In plants (16), animals such as the domestic dogs (17), and humans (18), when such lipid droplets have been characterized, they are commonly described as dynamic organelles with neutral lipids in the central core, surrounded by a monolayer of amphipathic lipids (phospholipids and cholesterol), with a distinct proteomic profile (19, 20). The lipid droplets are formed at microsomal (usually endoplasmic reticulum) membranes as primordial droplets with a diameter of 0.1–0.4 μm and increase in size by intracellular fusion. In skeletal muscle of athletes, intramuscular triacylglycerol-rich droplets are prevalent, despite being insulin-sensitive, as a cellular high-energy source (21). The kidneys, particularly RPTEC, of all animals are highly metabolically active (equivalent to the heart on a weight-specific basis) with high mitochondrial density (22). Thus, it is not beyond reason that the intracellular lipid droplets are related to cell metabolism and the local microenvironment, although such phenomena have rarely been described. An additional contribution toward propensity for renal lipid accumulation is suggested by biological sex; for example, in male cats, renal lipid content increases after castration or when sexual activity is lost in older age (8). In contrast, Martino-Costa et al. (9) concluded that interstitial lipid deposition affects male and female, neutered and non-neutered cats equally.

The precise composition of intracellular lipid droplets (LD), when characterized in other species appears to be organ, tissue, and cell-specific. For example, in liver, LD are described as comprising predominantly neutral, ether-soluble triacylglycerides, together with free or esterified sterols/cholesterol, surrounded by a phospholipid monolayer (18, 20). Whereas, in one *in vitro* study using a kidney cell line (normal rat kidney, [NRK]) in which LD were induced by dosing with oleate, LDs also accumulated modified lipids such as monoalk(en)yl-diacylglycerols (MADAGS) (23, 24). In felids, little is known about the composition of the prevalent LDs in kidney tissue. More extensive characterization may help to understand whether such LDs and the fatty acids that distinguish DCs from wild cats and dogs may be causally linked to the mechanisms of chronic interstitial nephritis (CIN) and CKD in cats.

Regardless, the presence of lipid droplets within cells of non-adipose tissue (i.e., ‘ectopic lipid’) is invariably associated with a chronic diseased/inflammatory state, such as obesity and/or organ fibrosis, through mechanisms linked to cellular lipotoxicity (1, 25–27). It is well known that both domestic (5, 28) and non-domestic felids (11), but not domestic dogs, have a propensity towards and indeed a high incidence of CKD. In young healthy cats, lipiduria and high concentrations of renal lipids remain an ‘incidental finding’. Any later causal association with feline CKD has not been proposed, despite the phenotype in cats being known for many years (8). Characterization of lipids (‘lipidomics’) is a complex science and many lipids remain unclassified. Esterified triacylglyceride is accepted as the common storage form of lipid (1); phospholipids (e.g., PC, PE) are structural components of biological membranes (29); hundreds of individual fatty acids exist in water-soluble, non-esterified form and participate in intracellular energy cycles’ (30). Some lipid classes are considered toxic when accumulated in various organs – diacylglycerides, ceramides and cholesterol (25, 31). Inter-organ exchange of lipid is a normal metabolic process and without consequence, unless lipids are deposited in the interstitium (i.e., tubulointerstitium in kidneys), where lipotoxicity elicits inflammatory reactions that underpin chronic interstitial nephritis/inflammation – a hallmark of CKD in felids (4, 9). However, no studies in companion animals have linked the common findings of renal intracellular lipid with CIN and CKD. Hence, further investigation of this phenomenon in cats is warranted.

In this study, renal lipids of DC (with or without CIN), domestic dogs (DD; as comparator domestic species not prone to CKD but sharing similar environment and refined food as domestic cats), and Scottish wildcats (SW; as a non-domestic felid, but eating unprocessed, unrefined food) were extracted, characterized, and compared from tissues of otherwise healthy cats (i.e., did not die or were euthanized for renal causes) or from cats with histopathologically diagnosed renal disease (e.g., diagnosed CKD). We hypothesize that: (1) DC kidneys contain more lipid than domestic dogs or SW, (2) the lipid content of the DC kidney increases during renal disease, and (3) the composition of RPTEC lipids in DC kidney is unique and underpins propensity to CKD in DC.

## Materials and methods

### Sample collection and preparation

Initial studies (ca. 2018–2020) characterized lipid droplets (e.g., by Oil Red-O, ‘ORO’) in kidney tissue from DC with histopathologically defined chronic interstitial nephritis (CIN; *n =* 13) or without renal disease (*n =* 12), and in DC (*n =* 12) and SW (*n =* 6). Sample details are described in Supplementary Information. All samples were obtained from Veterinary Pathology, Nottingham University School of Veterinary Medicine and Science. Ethical approval for use of pathological tissue was granted by the Committee for Animal Research and Ethics (CARE), University of Nottingham on a number of occasions (REC: 3256 201020; 3900 230809; 4055 240124). From 2021 to 2023, further samples were collected to increase the analysis to include chromatographic and mass-spectrometry studies. Further, DC (*n =* 21; 11 female, 9 male, 1 unknown, average 6.9 years) and domestic dogs (*n =* 7; 1 female, 6 male, average age 5.2 years). Of these, 9/10 cats were neutered, and 11 were of unknown status, compared to dogs where only 2/7 were neutered. Samples were obtained on an opportunistic basis. In addition, opportunistic samples from non-domestic feral wildcats were obtained from National Museums Scotland (SW [*Felis silvestris*]) (*n =* 18; 5 female, 13 male, 4 juveniles/kittens, 14 adults) and captive zoo wildcats (*n =* 3; all female: 18 years, 15 years, and unknown age). All kidneys were stored between −20 °C and −80 °C upon collection, and studies described herein were conducted between 1 month and 2 years post-collection. A typical workflow for sample characterization and lipid analyses is graphically presented in Supplementary Figure S1b.

### Histological evaluation and quantification of renal lipids

Hematoxylin and eosin stained FFPE kidney tissue sections (5 μm) were used to visualize gross pathology by light-microscopy (Nikon *i*50/80; Nikon Digital Sight DS-U1). Frozen kidney tissue sections (10 μm) from 21 DC, 22 dogs, and 8 SW were prepared onto polylysine slides at −20 °C for staining of tissue lipid using ORO. In brief, using three different samples of adipose tissue as a positive control, sections were immersed in 1% ORO in 70% industrial methylated spirit (IMS) for 30 min, further rinsed in 30% IMS, washed in running tap water, rinsed in distilled water, then counterstained with hematoxylin (30 s). Finally, after washing in tap water, blued sections were aqueous mounted and a coverslip applied. All sections were pseudoanonymised (e.g., N85-2345) before ORO quantification: 10 regions of interest in the cortex were assessed per section (random selection using a zig-zag construct) and images were saved. Image J was used to quantify greyscale images of percentage ORO positive areas (ORO^+ve^ – as proportion of total area with tissue). Imaging parameters (hue, saturation, and brightness) were initially optimized then fixed and applied to all sections. An average ORO^+ve^ area per slide was calculated.

### Extraction of renal intracytoplasmic lipid droplets

First a lipid extract was isolated from kidney tissue using the sucrose cushion method of lipid extraction by ultracentrifugation (Supplementary Figures S1a–m). A stock solution of isolation buffer (1 L) (50 mM HEPES (4-(2-hydroxyethyl)-1-piperazineethanesulfonic acid), 10 mM potassium chloride, 62.5 mM potassium acetate, 5 mM EGTA (ethylene-bis(oxyethylenenitrilo)tetracetic acid), 5 mM DTT (1,4-dithiothreitol), 1 mM magnesium chloride, and 5 mM EDTA; pH = 7.5) was prepared. Approximately 1 g of frozen renocortical tissue was homogenized on ice in 2 mL of 0.6 M protease-free sucrose (with 1% BSA) extraction buffer, using a gentleMACS™ tissue dissociator. The homogenate, with all cellular material including cell membrane lipids, was then centrifuged at 3,222 rpm (2,000 *g*) at 4 °C for 5 min, to remove cell debris from the cytoplasmic isolate containing cellular lipid. Further, 2 mL of this supernatant was overlayed on 1 mL 0.6 M protease-free sucrose extraction buffer and 2 mL 0.25 M protease-free sucrose (with 1% BSA) extraction buffer was overlayed on top to create a discontinuous sucrose cushion. The layered sample (in a 5 mL ultracentrifuge tube) was ultracentrifuged at 100,000 *g* for 1 h at 4 °C using Hitachi CP80NX, P55ST2 rotor. Cytoplasmic lipids (i.e., those not embedded in a bilayer membrane) are less dense than the sucrose solutions, which create a turbid layer that can be seen with the naked eye, at the top of the ultracentrifuge tube. These tubes were then frozen overnight at −20 °C and the top 1 cm of the ultracentrifuge tube was removed for further processing. This sample is referred as the total lipid extract (TLE; Supplementary Figure S1i). To 1 mL of the TLE, 5 mL of 2:1 (v/v) chloroform:methanol solution was added, vortexed twice (15 s) before adding 1 mL of 1% NaCl. Further vortexing ensured a homogenous lipid solution (soln. A) which was centrifuged (1,000 *g* for 2 min). The lower organic phase, comprising the lipids of interest, was transferred into a new glass vial (soln. B). Followed by adding 2 mL 100% chloroform to soln. A, vortexed twice for 15 s and further centrifuged at 1,000 *g* for 2 min. The lower organic layer was again transferred and pooled with soln B, before drying to completeness under nitrogen atmosphere, re-suspended in 200 μL 100% chloroform and stored in vials at −80 °C.

### Characterizing and describing the lipid extract

#### High performance thin layer chromatography (HPTLC)

**Figure 1:**
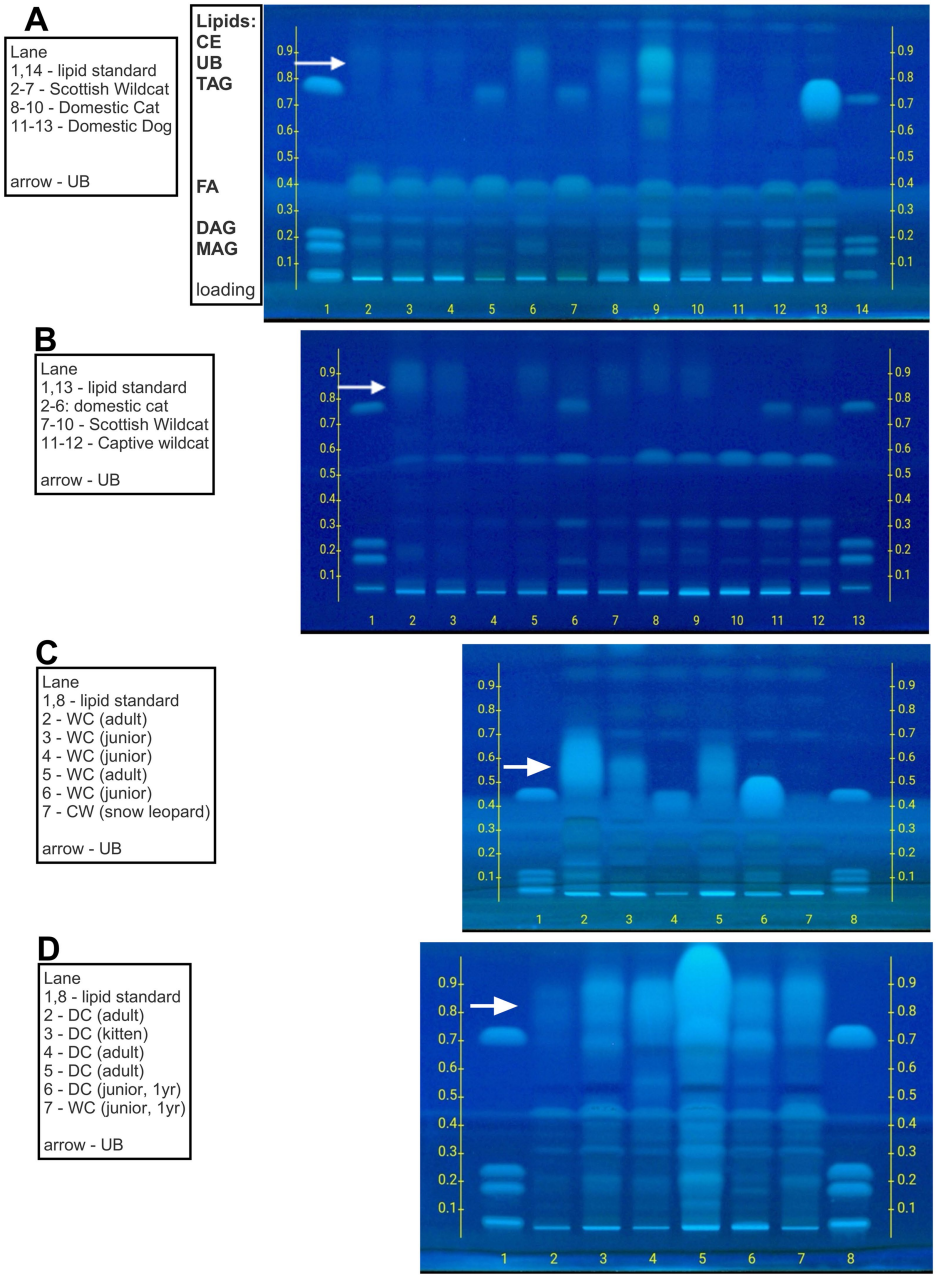
High Performance Thin Layer Chromatography (HPTLC) separation of major lipid classes in domestic cat (DC), Scottish wildcat (SW), Captive Wildcat (CW) and dog kidney after extraction and isolation of intracytoplasmic lipid droplets. **(A-D)** High Performance Thin Layer Chromatography (HPTLC) separation of major lipid classes in domestic cat (DC), Scottish wildcat (SW), Captive Wildcat (CW) and dog kidney. Each lane represents a separate sample with lipid standard (containing mono, di and triglyceride; [MAG, DAG and TAG]) run first and last on each plate. Further detail of each individual is given in Supplementary Information; Supplementary Item 2. Arrow indicates relative position of unidentified band (UB) on each plate. All unknown samples were loaded at 10µL, all lipid standards at 5µL. TLC carrier phase was hexane:diethyl ether:acetic acid (68:12:0.4 (v/v/v)).

The separation and identification of TLE lipids was performed using HPTLC. About 5–10 μL of the lipid standard mix containing 1,3-diolein, 1,2-dioleoyl-rac-glycerol, glyceryl trioleate, monoolein (1787-1AMP, Merck, United Kingdom), and 10 μL of TLE was applied on a HPTLC silica gel 60 F254 (20 × 10 cm) glass plate (Merck, United States) using a Linomat V (Camag, Switzerland) sample applicator. To maximize chromatographic resolution and facilitate superior analyte separation, the plate was developed twice in a chromatographic chamber (Camag, Switzerland) saturated with a mobile phase of hexane:diethyl ether:acetic acid (68:12:0.4 (v/v/v)). Once dry, the plate was immersed in a solution containing 10 mg primuline dissolved in 200 mL acetone:water mixture [80:20 (v/v)] using The CAMAG^®^ Chromatogram Immersion Device 3 (Camag, Switzerland) set at immersion speed ‘1’ and duration setting ‘6’. The plate was scanned RT White, at 254 nm and 366 nm using TLC Visualiser 2 (CAMAG, Switzerland). The bands of interest were marked in pencil (e.g., unidentified band or ‘UB’; Figure 1) and scraped for further analysis. Image profiles were generated, and data were processed using VisionCATS version 3.2 software (Camag, Switzerland).

#### Isolation of specific lipids from HPTLC bands of interest

For analysis of known or unknown lipids in specific bands on the TLC, the bands were scraped using a flat-end spatula and transferred to a 5 mL glass vial. In negative samples (i.e., no visible ‘UB’), a similar area was also scraped, for comparison. Further, 1 mL of 100% chloroform was added and gently agitated before heating at 35 °C for 20 min. Then remaining chloroform was transferred to a new vial (‘Vial A’), taking care to not include silica particles. The silica particles were treated with 1 mL of 100% chloroform, re-agitated for 15 s, and the resulting chloroform (excluding silica), was pooled together with ‘Vial A’ and frozen at −20 °C (Supplementary Figure S1a).

### FAME lipid extraction and separation

To the extracted lipid suspension, 0.7 mL 10 M KOH and 5.3 mL methanol were added. The sample was then heated at 55 °C for 90 min. After cooling, 0.58 mL 12 M H_2_S0_4_ was added and further incubated at 55 °C for 90 min. After cooling, 3 mL hexane was added, mixed, and centrifuged. The upper hexane layer was removed, concentrated by drying under nitrogen, and reconstituted in 400 μL hexane before storing at −30 °C until GC–MS analysis.

### Gas chromatography-flame ionization detection (GC-FID) of FAMEs

The fatty acid methyl esters (1 μL) were injected (split ratio 50:1) into a gas chromatograph (GC) (Trace 1300, Thermo Fisher Scientific™) coupled with mass spectrometer (MS) (ISQ 7000, Thermo Fisher Scientific™). Separation of FAMEs was performed with a Varian CP-Sil 88 (100 m length, 0.25 mm diameter, and 0.20 um film thickness, Agilent) using capillary column with helium as carrier gas. Oven temperature was programmed to ramp up at 4 °C/min, from 140 °C (held for 5 min) to 240 °C (held for 10 min). The MS injector and transfer line temperatures (260 °C and 250 °C, respectively) were preprogrammed. The ion source temperature was set to 200 °C. Characterization and identification of FAMEs was performed in scan mode, while quantification was completed by selective ion monitoring (SIM) mode of the most intense fragments. Data acquisition and processing were performed with Chromeleon (version 7.0, Thermo Fisher Scientific™). Quantification was based on external calibration with C19:0 as an internal standard.

### Liquid chromatography–mass spectrometry (LC–MS)

The lipidomics liquid chromatography (LC) method was based on that previously described (32). For LC, an Ultimate 3,000 HPLC (Thermo Scientific) was operated in reverse-phase mode with an ACE Excel 2 SuperC18 column (50 × 2.1 mm; 2 um particle size; Advanced Chromatography Technologies, Aberdeen, United Kingdom), maintained at 50 °C. The starting mobile phase was 30% B (10% water, 0.1% ammonium acetate, 10% acetonitrile, and 80% isopropanol) and 70% A (60% water, 0.1% ammonium acetate, and 40% acetonitrile) at a flow rate of 400 μL/min. The gradient was increased to 35% B by 1 min, then to 100% B by 7 min. The flow rate was then increased to 500 μL/min by 11 min. The proportion of mobile phase B decreased to 20% by 12 min, followed by equilibration for 3 min. For MS, the QExactive Plus Orbitrap (Thermo Fisher Scientific, Hemel Hempstead, United Kingdom) was used for LC–MS simultaneous ESI+ and ESI− modes. The probe and capillary temperatures were maintained at 412.5 and 256.25 °C, respectively. The following settings were used: sheath gas 47.5, auxiliary gas 11.25, and sweep gas 2.25, AGC target 3. The spray voltage was set to +4.0 kV or −4.0 kV. All files were acquired to get a full scan (70,000 resolution). Then data-dependent tandem MS/MS (ddMS^2^) spectra were produced on the five most intense ions at any one time at a resolution of 17,500, with collision energies at 20, 30 and 40 eV, isolation window 1.0 m/z, intensity threshold 1.6 10x^5^, and dynamic exclusion 8 s.

For later experiments, L-MS conditions were modified to achieve greater resolution and specificity. Mobile phases A and B remained unchanged, but after the starting ratio of 70% A and 30% B at a flow rate of 400 μL/min, the gradient was increased to 35% B by 1 min, to 100% B by 7 min, with the flow rate increased to 500 μL/min by 11 min and 600 μL/min by 12 min. Finally, the flow and gradient became 400 μL/min and 30% of B by 13 min, equilibrating at this level for 6 min for a total run time of 19 min. For the MS analysis, a Lumos Fusion Trihybrid instrument (Thermo Fisher Scientific, Hemel Hempstead, United Kingdom) was used. To enhance MS^2^ quality, data were acquired separately in positive and negative modes. The spray voltage was 3.5 kV for the positive mode and 2.5 kV for the negative mode. The sheath gas was 50, aux gas was 10, and sweep gas was 1, all in arbitrary units. Ion transfer and vaporiser temperatures were 325 and 350 °C, respectively. A predesigned workflow of General lipid profiling MS^2^ was adapted. The MS1 resolution was 120,000 with a scan range of 250–1,500 m/z. Data-dependent MS^2^ was performed with an isolation window of 1.5 m/z. Assisted HCD collision energies of 15, 30, and 45 eV were applied. Data acquisition was carried out at a resolution of 15,000 resolutions.

### *In situ* lipidomics of frozen kidney sections using OrbiSIMS

Orbitrap secondary ion mass spectrometry (OrbiSIMS) enables label-free imaging of tissues (33) and cataloging of complex biological sample chemistry (34). About, 10 μM sections of frozen kidney cortex from DC (*n =* 2), SW (*n =* 2), and captive wildcat (*n =* 2) were obtained using a cryotome (Leica Biosystems) at −20 °C, adhered to glass polylysine slides, and stored at −20 °C. Cryotome samples were transported on ice to the School of Pharmacy (University of Nottingham) for OrbiSIMS analysis. Slides were transferred into a Leica VCM bath under liquid nitrogen, attached to a Leica cryogenic block. Blocks were introduced into an airlock on the cryo-sample holder through a Leica vacuum transfer system. Analysis was carried out at −170 °C using a closed-loop liquid nitrogen pumping system (IONTOF GmbH). For acquisition of OrbiSIMS images, a 20 keV Ar3064+ analysis beam of 2 μm diameter with duty cycle set to 27% was used as primary beam, with a current at 24 pA. Obtained images represented an area of 400 μm × 400 μm using random raster mode. Pixel size was 4 μm, thus total number of pixels was 100 × 100 over a cycle time of 200 μs. Argon gas flooding was in operation in order to aid charge compensation, with pressure in the main chamber maintained at 9.0 × 10^−7^ bar. Spectra were collected in negative polarity, over a mass:charge (m/z) range of 75–1,125. Injection time was 500 ms, and mass-resolving power was 240,000 at 200 m/z.

### Data analysis and presentation

Data were collected using a multitude of analytical equipment and analysis software, as indicated in the main text. Data were primarily presented as raw data to show distinct differences between individuals. VisionCats (CAMAG) and Freestyle (Thermo Fisher Scientific) were used to visualize data. Lipid identification was completed using a combination of Lipidsearch 5.1(Thermo Fisher Scientific) and LIPID MAPS^®^. Quantitative data were processed and presented using GraphPad Prism v10.3.0 (GraphPad Software Inc., California, United States). Unpaired *t*-tests were used to compare OrbiSIMS groups.

### Data availability

Any data relevant to this manuscript are available from the authors on reasonable request. Lipidomic data are available from The University of Nottingham research data repository^1^.

## Results

### Oil Red-O staining of kidney tissue

**Figure 2:**
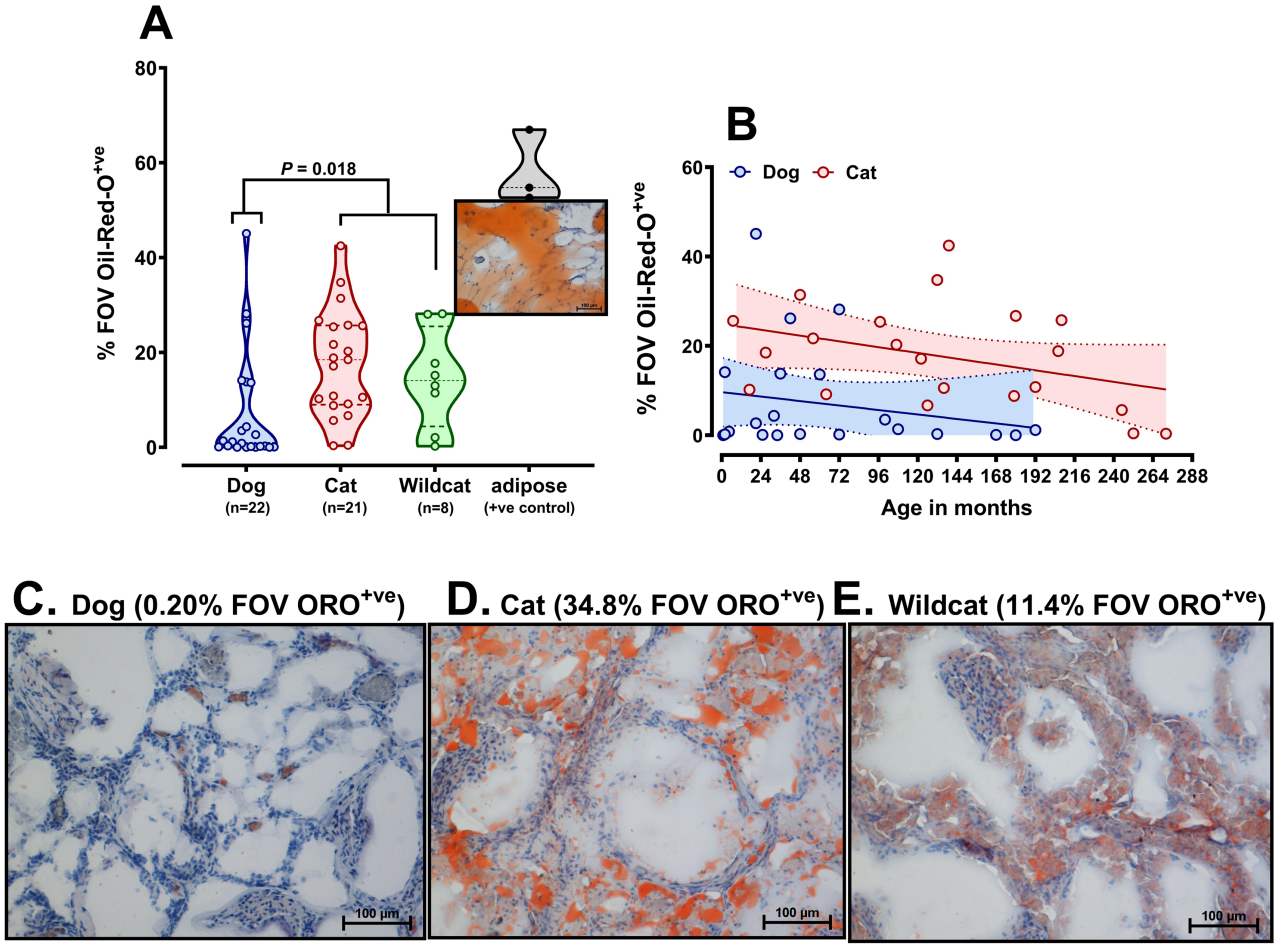
Oil-Red-O (ORO) staining of lipid in domestic cat, Scottish wildcat and dog kidney sections. **(A)** Violin plot of individual samples (represented as circles) of domestic cat, Scottish wildcat and dog kidney sections, stained with Oil Red O, using adipose tissue as a positive control. Data are average of ten random fields of view stained positively with Oil Red O per slide. The full range of data is depicted within the violin plot, dashed lines representing the lower and upper quartiles, with median. Lipid content of cat and wildcat kidneys were similar, each being significantly greater than dogs (*P* = 0.018, NP-ANOVA). **(B)** Scatter graph of percent ORO^+ve^ versus age of individual. Solid lines are lines of best fit, dotted lines indicate 95% confidence interval around the mean for age. **(C-E)** Representative images of ORO stained kidney sections from **(C)** dog, **(D)** domestic cat and **(E)** Scottish wildcat. Images were taken with a Nikon Digital Sight DS-U1 camera, Nikon i80 light microscope, 20x objective.

The proportion of kidney tissue positively stained with Oil Red-O (ORO^+ve^) was similar between DC and Scottish wildcats, but each had significantly greater lipid content than the DD (DC, 18.48% [9.069, 25.66%]; Scottish wildcats, 14.09% [6.756, 22.90%]; dogs, 1.002% [0.0917, 13.63%], median [IQR]; *p* = 0.018) (Figures 2A,C). As a positive control and for reference, sections (*n =* 3) of adipose tissue were 54.76% [53.18, 63.92%] ORO^+ve^, as expected (Figure 2A). At all ages available, DC had greater lipid content than dogs (Figure 2B), even in the few samples available ≤2 years of age. The intercept of the lines of best fit for cats and dogs was significantly different by linear regression (*p* = <0.001). Over time, in both species, it was apparent that the ORO^+ve^ lipid content of kidneys gradually declined rather than increased (Figure 2B), likely reflecting the age-related loss of RPTECs, which primarily harbor lipid droplets in feline kidneys. Next, this study sought to characterize and describe the identity of the prevalent cytoplasmic lipids in felids (cf. dog), employing multiple lipidomic approaches (as summarized in Supplementary Figure S1b).

### High performance thin layer chromatography (HPTLC)

Total lipid extracts (TLE) separated into major lipid classes by HPTLC using 24 DC, 12 domestic dogs, 6 Scottish wildcats (SW), and 3 captive wildcats (CW), indicated an unusual, unidentified lipid class (hereafter referred to as ‘unidentified band’ or UB) in the majority of DC (i.e., 23 of 24). The lipid was less polar than triacylglycerols (TAG), the predominant lipid present in most samples, and was more polar than cholesterol esters (CE; see Figures 1A–D). Further analyses confirmed the presence of UB in all adult DC kidney samples, whether healthy (*n =* 12) or DC with a previous diagnosis of CIN (*n =* 12), the defining histopathophysiological feature of CKD in DC.

Indeed, further analyses of TLE by HPTLC from an additional sample set (18 DC, 7 domestic dogs, 21 SW, and 3 CW) confirmed previous observations; no canine or CW (*n* = 3 including 2 snow leopards and 1 tiger; Figure 1B) exhibited a UB. The UB was occasionally observed in adult SW (Figure 1C). Most TLEs contained common lipids present in the reference, e.g., monoalkylglycerol (MAG), 1,3-diacylglycerol and 2,3-diacylglycerol (DAG), free fatty acids (FA), and triacyclglycerol (TAG). The UB eluted at an Rf value of ~0.9–1.0, between TAG and CE, and often appeared to substitute for TAG, but this was dependent on age. For example, in kittens only the TAG band was present; whereas, in elderly individuals the TAG band diminished but the UB became more prominent. In CW, only the TAG band was prominent (Figures 1C,D). Semi-quantitative evaluation of this second group of samples using HPTLC chromatograms showed that the ratio of TAG: UB varied with age: in kittens, TAG: UB was 22.6:20.0% versus adult DC, where the ratio was 6.4:25.3%. SW were similar (kittens, 24.1:14.0% versus adults, 7.8:21.2%). Dogs and captive zoo wildcats were consistently ~15.1–16.1% TAG but negative for the UB. Thus, extraction of intracytoplasmic lipid from various felids, using domestic dogs as a domestic comparator/reference species, revealed the virtually exclusive presence of an unidentified lipid band in DC. The band appeared to share many characteristics of TAGs but is less polar. This finding suggests modifications of the fatty acyl chains of TAG (e.g., ether or alkyl bonds are less polar than ester bonds). The presence of such ‘modified TAGs’ remains less common (at least in our hands) in kittens or other young felids, despite renal lipid droplets being previously described at this age (Figure 2B). The UB appeared to become more prevalent with age, often substituting/replacing TAG at this time. We therefore further characterized the UB in terms of fatty acid composition (to suggest the types of fats present) and by employing mass-spectroscopy-based lipidomics (to directly identify the lipid species present in the TLE and in the individual bands).

### Liquid chromatography mass spectrometry of total lipid extract (TLE) from kidneys

**Figure 3:**
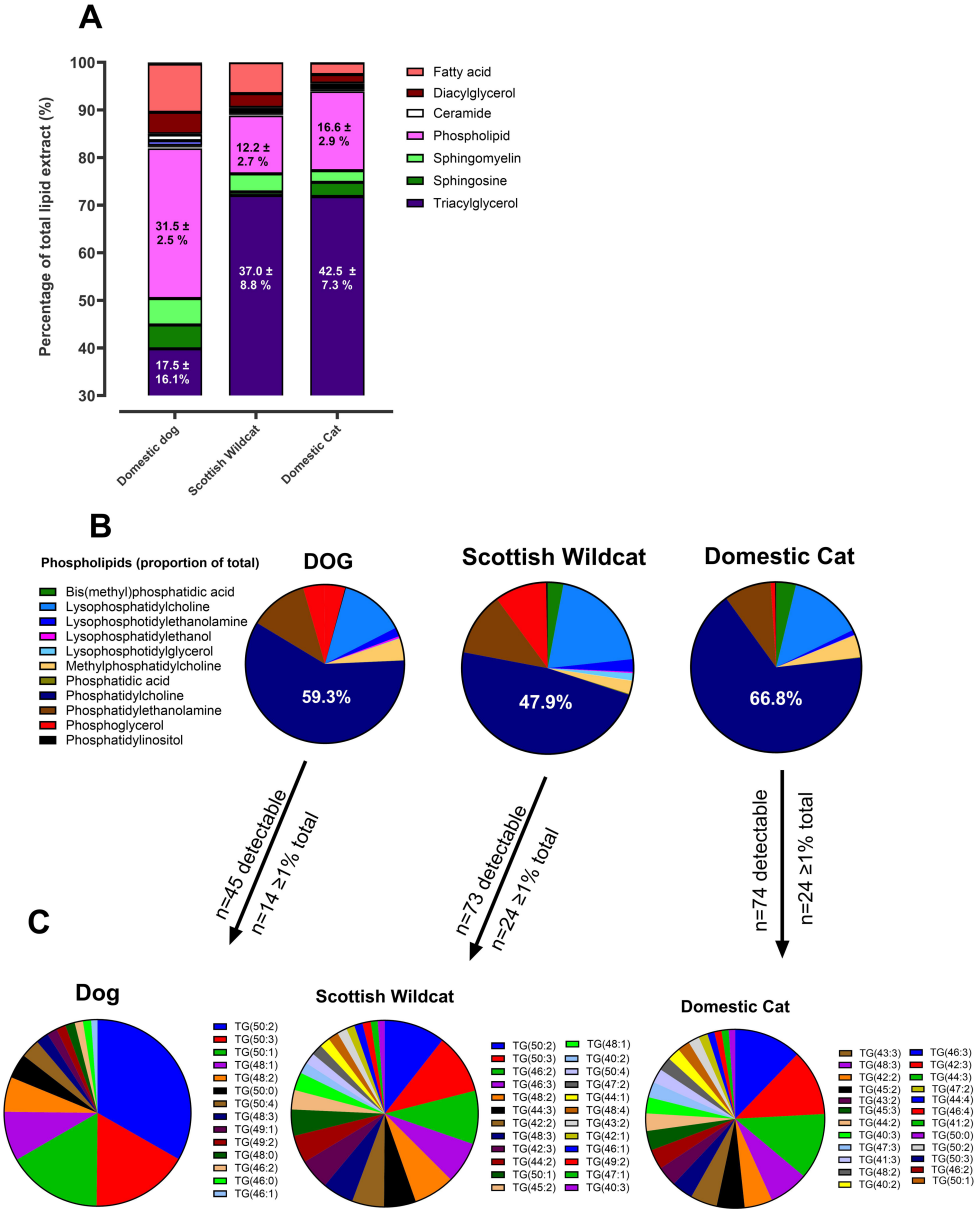
LC-MS analysis of total lipid extract (TLE) in dog, Scottish wildcat and domestic cat kidneys. **(A)** Percentage composition of lipids in domestic dog (*n* = 4), Scottish wildcat (*n* = 6) and domestic cat (*n* = 9) total lipid extract by percentage of total, as determined by LC-MS. Average group (as determined by Lipidsearch) composition of dog, SW and DC **(B)** phospholipids and **(C)** triacylglycerols >1% of total.

Lipid extracts from 19 individuals (9 DC, 4 domestic dogs, and 6 SW) were assessed by LC–MS, with annotation of lipid classes using Lipidsearch™. A total of 2,353 different lipids were identified. As expected, SW and DC had a greater proportion of TAG relative to dog (DC, 71.6; SW, 71.8 vs. dog, 39.4%; Figure 3A). Phospholipids were the other major contributors to the lipids in TLE between species (Figure 3B). For example, the dog TLE comprised 31.5% phospholipid (versus DC, 16.7% and SW, 12.2%), primarily comprised of phosphatidylcholine (59.3%, colored purple in Figure 3B) and lysophosphatidylcholine (14.3%, blue). DC and SW were broadly similar in overall profile, although DC had greater phosphatidylcholine (66.8%), while SW had greater phosphoglycerol (9.8%) relative to DC (colored red in Figure 3B). Thus, companion animal kidneys are highly enriched in neutral lipids; beyond TAG and phospholipids, no other lipid contributed >3%. Characterizing the TG species (numerous, Figure 3C) revealed differences between dog and felids: dogs had fewer species, dominated by three individual TGs (50:2, 50:3, 50:1), whereas felids (DC and SW) had far greater number of detectable species, but were broadly similar between DC and SW (Figure 3C).

**Figure 4:**
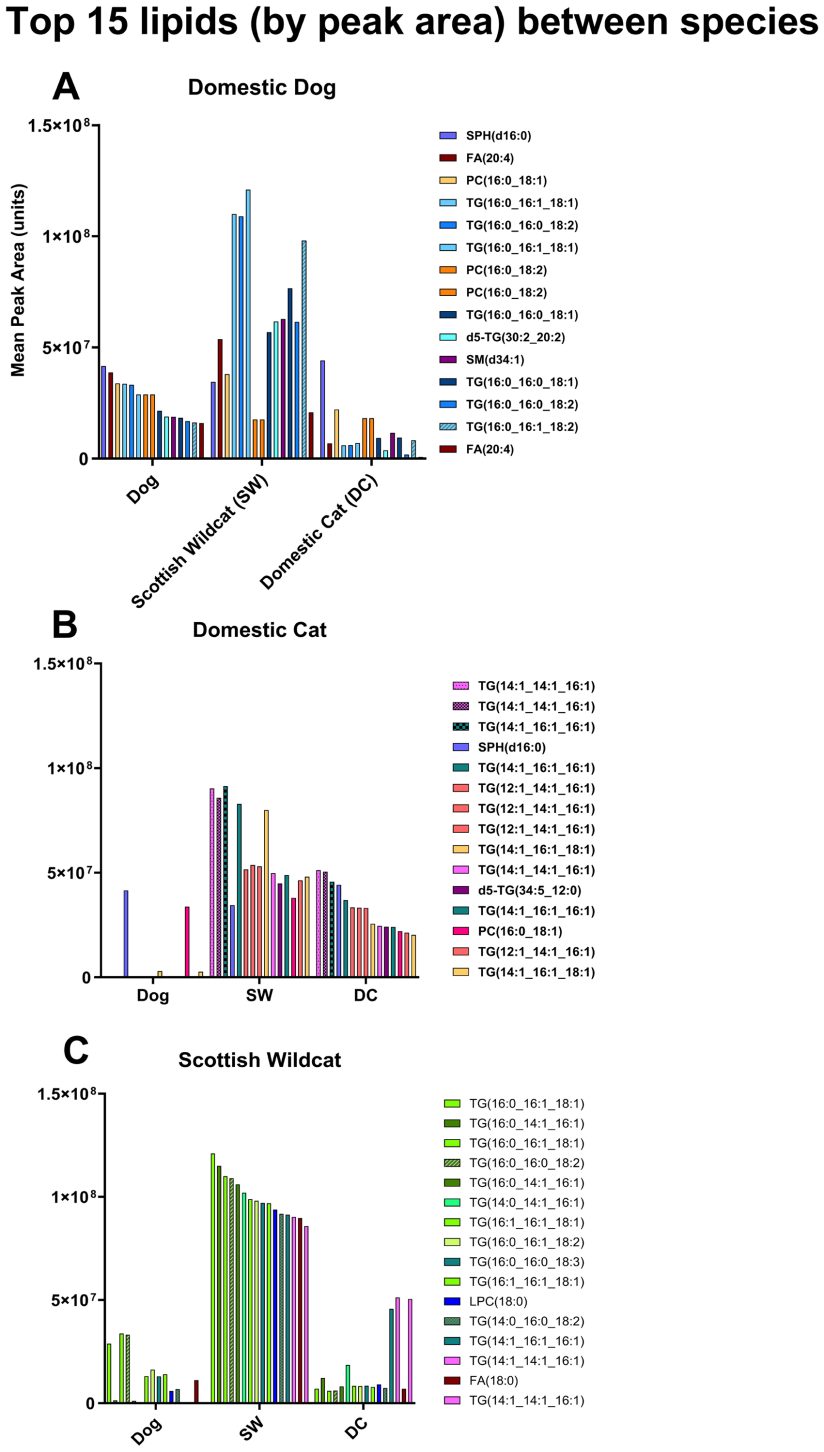
Top 15 lipids extracted from kidneys (Dog, Domestic cat, Scottish Wildcat). Top 15 lipids detected in renal cortical tissue of **(A)** domestic dog (‘DOG’, *n* = 15), **(B)** domestic cat (‘DC’, *n* = 16) and **(C)** Scottish Wildcat (‘SW’, *n* = 16). Bars represent mean peak area for each lipid across all individuals of that species. Each colour corresponds to a specific lipid identity, and is consistent across **(A-C)**. While some lipids are shared between species, the top 15 lipids from each species are largely dominated by different lipids.

Comparing the 15 most abundant lipids extracted from each species kidney, DC was almost exclusively comprised of shorter, mono-unsaturated fatty acids such as TG(14:1_14:1_16:1) and TG(14:1_16:1_16:1), also present in SW but undetectable in domestic dog (Figures 4A,B). In the latter, combinations of common saturated fatty acids were dominant such as palmitic (16:0), oleic (18:1), and linoleic (18:2) acid [e.g., TG(16:0_16:1_18:1) and TG(16:0_16:0_18:2), Figure 4A]. In SW, the top 15 lipids were also present in DC, but at much lower levels {e.g., only one of the top 15 also appeared in DC; [TG(14:0_14:1_16:1)] and were virtually absent from domestic dog (Figure 4C)}. Thus, felids have more TAG in their kidney cortex than dogs, with a much greater phosphatidylcholine to total TAG ratio, and far greater TG species – for felids. These TGs appear to have an unusually high proportion of short-medium chain, mono-unsaturated FA. Such differences could, partially, affect polarity and may explain the polarity shift from TAG to UB, by TLC. We therefore specifically isolated the UB (i.e., by scraping and extracting from the TLC plate) and characterize the individual lipids in the UB (cf. total lipid extract) by mass-spectrometry-based lipidomics and derivation of fatty acid methyl esters.

### Fatty acid methyl esters (FAMEs) in the UB

**Figure 5:**
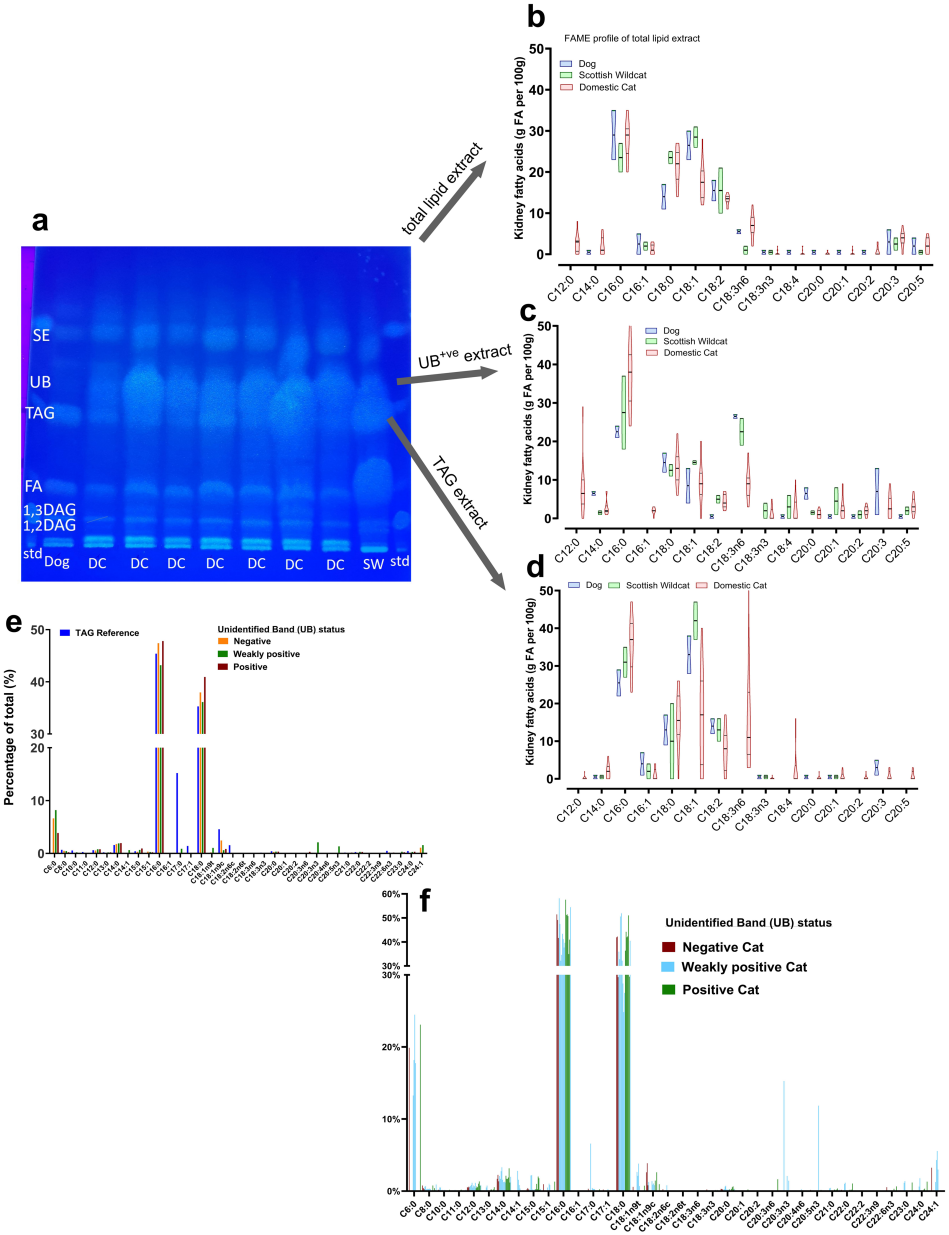
Fatty acid methyl esters (FAME) in domestic cat, dog and Scottish Wildcat kidney extracts **(A-D)**. **(A)** Example TLC plate of separate lipid classes (DC, domestic cat; SW, Scottish Wildcat; FA, fatty acid band, TAG, tryacylglycerides; UB, unidentified band; SE, sterol esters). Each band was scraped and FAME determined from **(B)** total lipid extract, **(C)** UB+ve band and **(D)** TAG band. **(E)** data are mean percent of total for FAME from further samples categorised specifically by UB status (UB^-ve^, UB^+ve^ and weakly UB^+ve^), **(F)** data are individual values for percent of each FAME for *n* = 18 domestic cats only, with all individuals separated by UB status.

Individual lanes on TLCs from felids were categorized as either positive (UB^+ve^) or negative (UB^−ve^) for visual presence of the unidentified band (see example, Figure 5a). The area was visualized, scraped, and extracted appropriately for analysis of FAME content (Figures 5b–d). A similar process was adopted for various TAG standards, to confirm recovery of expected fatty acids (e.g., stearic, C16:0 and palmitic, C18:0). In most samples from total lipid extracts and the TAG band, the majority fatty acids were, as expected, C16:0 and C18:0 series (Figures 5b,d). Notably, in the UB^+ve^ band – largely exclusive to DC – C12:0 (lauric acid) and C14:0 (myristic acid) were common, whereas in dogs it was absent (Figure 5c). Nevertheless, in the protocol for methylating FAMES, an ester bond is cleaved in exchange for an alcohol functional group. Thus, it is perhaps unsurprising that further FAME analysis did not reveal any marked/notable differences between the species, beyond a preponderance of short-to-medium chain FA in DC (such as caproic, [C6:0]; lauric [C12:0], and myristic [C14:0], Figures 5e,f). FAMES, which derivatives lipids with ester bonds, would not report difference in lipids with potential ether bonds, which would reduce their polarity due to addition of a hydroxyl group, be comprised of similar fatty acids, but migrate further up a TLC plate. Indeed, although rare in mammals, such ether-soluble neutral lipids (with or without alkyl chains), are stored in lipid droplets (24) and migrate to a similar location on TLC, as in our study (35–37). We therefore undertook an extended LC-MS/MS of the UB, in order to describe in detail the types of lipids found at this position.

### LC-MS/MS evaluation of the unidentified band

**Figure 6:**
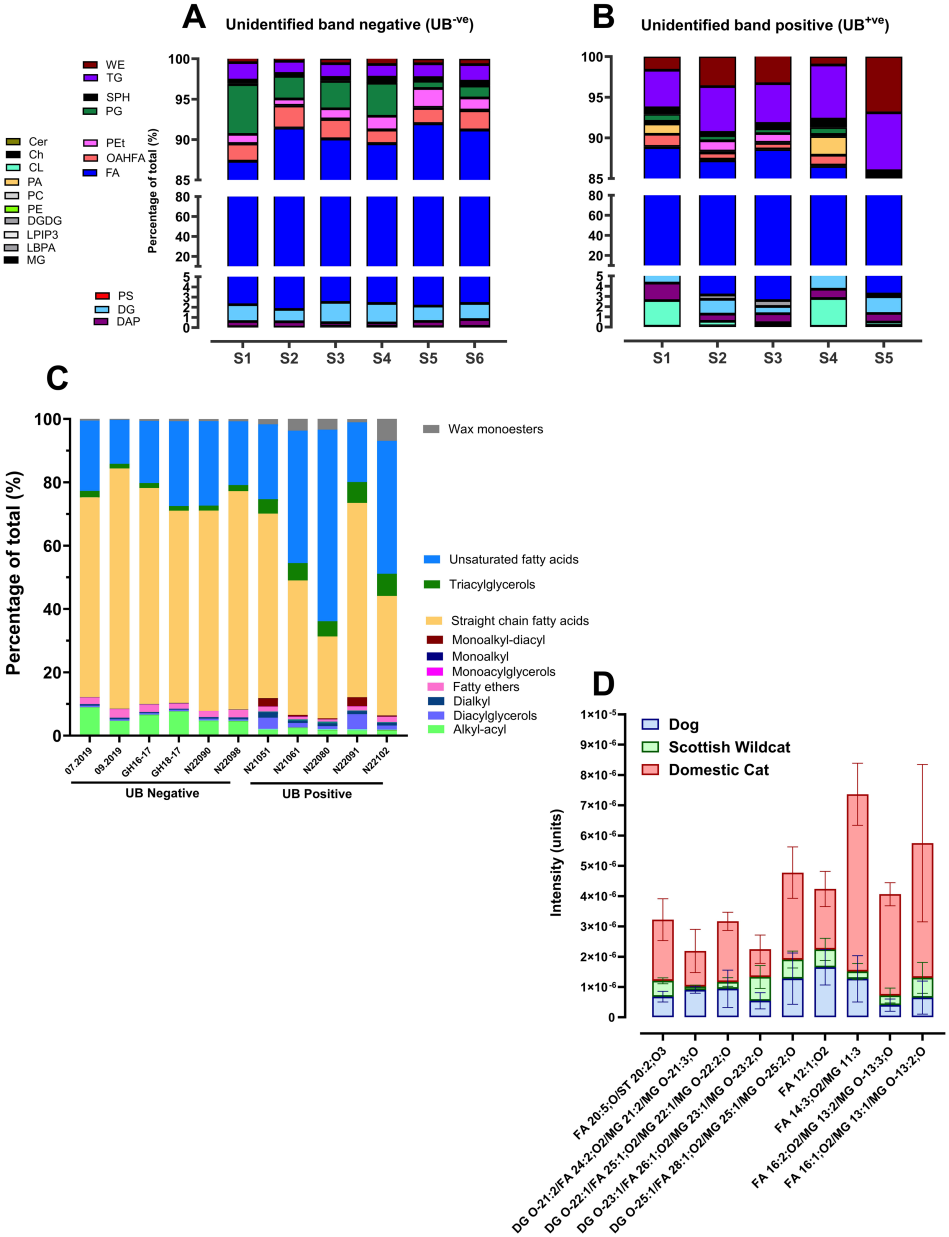
Lipid composition of unidentified band positive versus negative (UB^+ve^ versus UB^-ve^) in Domestic cat and Scottish Wildcat kidney extracts or frozen sections. Lipids determined by LC-MS or cryo-orbiSIMS. Relative percentages of lipid classes present in the unidentified band (UB) across **(A)** *n* = 6 UB^-ve^ and **(B)** *n* = 5 UB^+ve^ individuals, as determined by HPTLC. Cer: ceramide, Ch: cholesterol, CL: cardiolipin, DAP: diacylglycerol pyrophosphate, DG: diacylglycerol, DGDG: digalatosyldiacylglycerol, FA: fatty acid, LPIP3: lysophosphatidylinositol 3-phosphate, LBPA: lysobisphosphatidic acid, MG: monoacylglycerols, OAHFA: (O-acyl)-1-hydroxy fatty acid, PA: phosphatidic acid, PC: phosphatidylcholine, PE: phosphatidylethanolamine, PEt: phosphatidylethanol, PG: phosphatidylglycerol, PS: phosphatidylserine, SPH: sphingosine, TG: triacylglycerol, WE: wax ester. **(C)** Individuals from **(A)**, with percentages of lipid subclass instead, highlighting the distribution of fatty acids into unsaturated fatty acids and straight chain fatty acids. **(D)** Distribution of lipids in frozen kidney from *n* = 4 dog, Scottish wildcat and domestic cat, as determined by cryo-orbiSIMS.

Following extraction and purification of the UB and LC-MS/MS analysis, individuals were categorized according to their TLC result: with (UB^+ve^) or without (UB^−ve^) the UB. Fatty acids were the predominant lipid class in both groups (Figures 6A,B), but UB^+ve^ samples had much greater content of triacylglycerols (‘TG’) and wax esters (WE, Figure 6B). UB^−ve^ samples consistently had a greater proportion of phospholipids, particularly PG (Figure 6A). Expansion of the lipid classes into subclasses using Lipidsearch™, then the composition of the UB^−ve^ samples was relatively uniform; predominantly straight chain fatty acids (average: 66.43% [62.84–75.74%], Figure 6C), with relatively greater alkyl-acyl moieties (at ~6.00%) and fatty ethers (2.18%). In the UB^+ve^ samples these same classes were only 44.65% of total (straight-chain FA), 1.70% (alkyl-acyl lipids), and 1.16% (fatty ethers), respectively. In addition, the majority of UB^+ve^ samples had greater content of wax monoesters, unsaturated fatty acids, and triacylglycerols, as annotated by Lipidsearch™. Interestingly, of note, ‘monoalkyl-diacyl’ glycerols were noted in all UB^+ve^, but not UB^−ve^ samples, albeit at low levels (Figure 6C). Furthermore, LC-MS revealed that UB^+ve^ extracts were enriched in lipids with atypical compositions and mass profiles, including [O-31:2_23:2], [O-38:7], and [O-4:0_22:6], the ‘O-’ prefix indicates the presence of an ether linkage (38). Such ether-soluble, neutral lipids, particularly those with higher mass values that were present in all UB^+ve^ sample had similar m/z ratio and molecular formula (see Supplementary Table S1), as the MADAGs directly identified by Bartz et al. (23). Thus, DC samples that were UB^+ve^, in contrast to UB^−ve^ samples consistently had structurally distinct, non-traditional TAG analogs, potentially incorporating ether or branched-chain moieties. We then sought to confirm *in situ*, the presence of such unusual lipids in an unbiased, yet direct, analysis of frozen felid kidney sections with known tubular lipidosis.

Cryo-OrbiSIMS yielded a spectra of lipids overexpressed in frozen sections of renal cortex (one section per individual: *n =* 4 different samples of dog, DC, and SW) from felids verses dog (Figure 6D; Supplementary Figures S2–S5; Supplementary Table S2). From the 2,212 peaks detected, OrbiSIMS detected 733 peaks to be unique to domestic cats (cf. SW and dog) and of those peaks, LipidMaps™ returned 347 lipid assignments (see Supplementary Item 4). Many lipids had multiple identifications, having the same elemental composition, e.g., mass: 757.539 (C_42_H_78_O_9_P-) may be identified as PA 39:2; O or PG O-36:3. In this study, the initial annotations are presented. Lipid classes over-expressed in DC, included ‘branched-chain fatty acids’, mono-alkylglycerols, and ‘1-alkyl,2-acylglycerophosphoglycerols’(i.e., MADAGS; Figure 6D and Supplementary Figures S2–S5).

## Discussion

This study describes, for the first time, a comprehensive analysis of lipid content of kidney tissue commonly found in companion felids, with the dog as a referent category species for the domestic environment, and the Scottish wildcat as a non-domestic referent species. Using Oil-Red-O histochemistry, HPTLC, UPLC–MS/MS, GC-FAME, and cryo-OrbiSIMS, we have identified and described a relatively low-abundance, yet common for DC, unique profile of renal lipids. Felids in general have abundant Oil-Red-O positive (ORO^+ve^) neutral lipids encapsulated in intracytoplasmic lipid droplets of varying size, usually limited to renal proximal tubule epithelial cells, as previously described (14). Further analysis of these lipid droplets revealed sub-cellular components containing neutral triacylglycerides (~72% of total in felids, 39% in dogs), phospholipids (~17% of total in felids, 32% in dogs), and cholesterol, as expected (15). However, we additionally report, for the first time in a companion animal species, that DC commonly elutes a reproducible, lower-polarity (cf. TAGs) lipid band that was only occasionally present (albeit weakly) in Scottish wildcats, and was absent in dogs and most captive wildcats. The TAG-to-phospholipid ratio in DC is consistent with the presence of larger intracytoplasmic lipid droplets formed with a monolayer of phospholipids (mostly phosphatidylcholine). Further lipidomics (LC–MS and spatial cryo-orbiSIMS) and FAME analysis of DC lipid droplets and the unusual lipid band revealed the presence of a panoply of modified TAGs, such as odd- and short-chain fatty-acids, branched-chain fatty acids, and monoalkyldiacylphospholipids (MADAGS). All modified TAGs are less polar, by virtue of an ether-linkage, which accounts for the unusual banding on lipid chromatography. These data are highly unusual for mammals, particularly when one considers that DC show the appearance of renal lipid droplets from an early age. Taken together, the data suggest DC kidney commonly forms unusually modified (ether-linked and/or branched) neutral lipids (such as MADAGs or similar). Definitive structural analysis using reverse-phase HPLC together with NMR would confirm lipid identities. Regardless, given the known association of ectopic lipid, including lipid droplets, with organ fibrosis, and the known propensity for renal fibrosis in DC (28), we propose that the propensity for felids to develop tubular lipuria is one of the first pathophysiological outcomes connecting felid biology, diet, age, and vulnerability to chronic renal disease.

Evidenced by Oil Red-O and HPTLC, this study confirms that most felids accumulate lipids in their renal cortices, mainly the RPTEC from an early age (6–8, 15, 39). Expanding on these studies in cats, we can also report that the lipid droplet lipidome in cats is similar in many respects to experimental animal species; that is, the lipidome is comprised of triacylglycerides, phospholipids, and cholesterol (24). A full lipid profile of the cat’s renal intracellular lipid droplets has not been completed previously. Oil Red-O was used to stain neutral lipids, including triacylglycerides and diacylglycerides (40), as well as cholesterol esters (41). Analysis of total lipid in felid kidneys indicated that Scottish wildcats, like DCs, have evident lipids with a highly variable profile within their kidneys, despite less prevalent histopathologically determined lipid droplets. Thus, the presence of lipid in the kidney cortex appears common among felids. However, gradual bioaccumulation with age does not appear to be the case (see Figure 2), consistent with felids regularly exocytosing such droplets into tubular fluid (urine), perhaps explaining the common clinical pathological finding of lipuria in felids, whether domestic (42) or wild (10). Regardless, one feature of the lipids in kidneys of DC that is unique is the consistent presence of unusual lipid classes in the droplets, rarely observed in other mammals (37), but commonly observed in certain mollusks or squid (43) or in certain conditions of metabolic (i.e., hepatic) stress leading to significant lipolysis (44).

### Felid kidneys have an unusually common presence of rarely observed lipids

A distinct lipid band identified by HPTLC as having lower polarity than triacylglycerol and consistently observed in total lipid extracts from DCs (referred to as the unidentified band) was an unusual and unexpected finding. The band was absent from extracts of dog kidneys, only faintly present in some Scottish wildcats, and appeared unrelated to disease status; kittens, young adults, healthy adult felids with no renal disease, or those with diagnosed CKD all generally presented with a UB. Previous studies in other experimental species or under certain conditions have reported an identical band as being specifically, MADAGs (23, 37); that is, a TAG where a long-chain fatty alkyl group is connected to the *sn*-1 carbon of glycerol through an ether bond (36). MADAGs are essentially storage lipids and ether-analogs of triacylglycerols, and previously have only been noted in any quantity in cells within lipid droplets (24) or where inborn errors of metabolism lead to lysosomal (e.g., Wolman’s) disease (45). Quite why felids, particularly DCs, may be prone to accumulating MADAGs in lipid droplets in the kidney remains unknown and is beyond the scope of this study, but this information now opens up new avenues of investigation. Are the droplets formed *de novo* with MADAGs coming from diet? Ether lipids are more abundant in squid or oily fish species (46) – the by-products of which are often used to flavor DC feeds. However, given the prevalence of MADAGs in the majority of DC in the present study, and the fact that they are only observed in tissues with extensive peroxisomes and lysosomes (i.e., they are absent from adipose tissue), this suggests formation is local and, in part, due to some aspect of the local cellular microenvironment. Genetic analysis of DC suggested evolutionary adaptation to a diet rich in protein and fat – many over-expressed genes were associated with lipid handling (47). As ether lipids are either formed and/or broken down by specialized peroxisomal enzymes and other lipases involving mitochondria and endoplasmic reticulum, and the kidneys are highly metabolic organs (similar to heart), suggests that some aspect of felid kidney metabolism lends itself to inappropriate formation of intracellular lipid droplets (4).

The abundant lipids in DC were also enriched with short, mono- and polyunsaturated TAGs such as TG(14:1_14:1_16:1), many of which were completely absent or only minimally present in dogs – which processed TAGs mostly enriched with palmitic (16:0), oleic (18:1), and linoleic (18:2) acids [e.g., TG(16:0_16:1_18:1) and TG(16:0_16:0_18:2)]. Again, such an unusual profile of fatty acids has not been reported before in DC. The profile may be feline-specific, reflecting some peculiarity of fatty acid synthesis and turnover in the felid kidney, or diet – reflecting intake of a wide variety of different fats, or again, some aspect of felid kidney metabolism. Notably, consistent with previous findings in feral cats (48), the profile of fatty acids in Scottish wildcats were also much broader than that of dogs, but primarily comprised longer-chain FA combinations compared to DC (e.g., 16:0, 18:1, 18:2n-6, and 18:3n-3).

In addition, unidentified band-positive samples consistently had higher proportions of wax monoesters, and further sub-division elicited a transition from primarily straight-chain saturated fatty acids in UB^−ve^ extracts to unsaturated fatty acids in UB^+ve^. This suggests a compositional shift in neutral lipid content; specifically, UB^+ve^ samples had a higher proportion of ether-linked lipids such as [O-31:2_23:2], [O-38:7], and [O-4:0_22:6], as well as fatty acid C18:1 (oleic acid). Further confirmation that UB^+ve^ samples tend to be comprised of ether lipids comes from derivation of the fatty acids using methylation, i.e., analysis of FAMEs data: the protocol for preparation of FAMEs involves cleaving ester but not ether bonds. Both UB^+ve^ and UB^−ve^ extracts had broadly similar percent C16:0 and C18:0 (of total FAMEs). Odd-chain fatty acids (C15:0 and C17:0) were marginally increased in UB^+ve^ samples. This suggests that greater differences in fatty acids between felids with or without an UB is likely to involve non-esterified lipids. This hypothesis is further supported by slightly elevated levels of odd-chain saturated fatty acids, including tridecanoic acid (C13:0) and pentadecanoic acid (C15:0) in UB^+ve^ individuals. The presence of these odd-chain lipids in felid kidneys could indicate higher intake of branched-chain amino or fatty acids (BCFAs) in those felids that are UB^+ve^ or, as is more likely, *de novo* accumulation. The latter can occur when cells (usually liver) become depleted in certain micronutrients or excessive in others, such as the short-chain fatty acids (propionate and valerate). For example, in ovine white liver disease (a condition affecting sheep caused by cobalt deficiency, leading to vitamin B12 deficiency), the liver accumulates increased levels of branched-chain fatty acids, particularly propionate, due to disruptions in the metabolic pathway that utilizes these fatty acids, resulting in a characteristic pale, fatty liver (49). The extent to which felids may exhibit a similar cellular microenvironment, from an early age, is not known.

Here, direct, unbiased and non-destructive analysis of renal cortical tissue using cryo-orbiSIMS provided further support for a species-specific lipid signature in kidneys. Kidneys of DC had 733 unique peaks compared to dog and wildcat, 347 of which were assigned lipid identities. Among these were elevated levels of fatty acids, diacylglycerols, and monoacylglycerols – many of which were ether-linked (O-, see Supplementary Item 4). Indeed, *n =* 19 had accurate masses previously positively identified as MADAGS (23). The presence of differing lipids classes in lipid droplets of the same feline kidney tissue further suggests heterogeneity in lipid storage, potentially reflecting cell-specific lipid metabolism or sequestration, not surprisingly given the heterogeneity of cell types in kidney cortex. The distinct spatial distribution, combined with ether-linked phospholipid detection, reinforces the idea that feline kidneys may preferentially accumulate atypical lipid structures as either an adaptive or pathological response to chronic metabolic stress.

### Age and sex of the animal

Age was an important consideration in this study, as a more lipidic phenotype emerged from kitten through adult to senior cats, as previously observed (14). While the phenotype of tubular lipidosis is often macroscopically indistinct (8), the propensity to form lipid droplets appears to increase with age – 100% of cats ≥15 yrs. had tubular lipidosis, according to Quimby et al. (14). We observed the overall fatty acid profile of young and senior cat kidneys to be similar, but the propensity for TAG to migrate and become UB^+ve^ was notable; young cats generally had a prominent TAG band with sometimes a weak UB^+ve^ or ‘MADAG’ band. In contrast, for senior cats, the presence of a prominent UB^+ve^ or ‘MADAG’ band tended to be associated with a weak TAG band; our interpretation being that with seniority, the renal cells become more predisposed to the conditions that exacerbate the formation of intracellular lipid droplets with a unique lipid signature. The two oldest cats in this study were 13-years and 15-years of age females, both were UB^+ve^. In contrast, a male Scottish wildcat kitten and a 7-month-old female were both UB^−ve^. Regardless, it is accepted across animal models, experimental or human, deposition of ectopic lipid (i.e., lipid not stored in adipose tissue) is toxic to cells, including renal cells (31, 50). The relatively high lipid content of the domestic cat kidney, evidenced as early as young adulthood, whereas first stage symptoms of renal disease do not become evident until approximately 7–8 years of age, would suggest a role for tubular lipidosis in the aetiopathogenesis of CKD in felids, a hitherto unforeseen and overlooked cause as yet.

In summary, felids appear inherently prone to forming intracellular lipid droplets in their metabolically active renal cells from an early age. In DC, these lipid droplets exhibit a unique lipidomic signature, characterized by fatty acids (e.g., odd-chain, branched) and lipids (e.g., MADAGS) that are rarely seen in mammals, likely reflecting the local cellular microenvironment. Although, lipiduria has been commonly referred to as ‘incidental’ in DC, further work should determine whether the lipids in feline urine are the same as those found in lipid droplets in kidney cells (i.e., reflecting regular turnover). Regardless, ectopic lipid deposition is always deleterious. We therefore purpose that tubular lipidosis may represent as one of the earliest indicators of a renal cellular environment that, over time, manifests to CKD.

## Data Availability

The data presented in the study are deposited in The University of Nottingham research data repository with accession number http://doi.org/10.17639/nott.7634.

## Footnotes

1
http://doi.org/10.17639/nott.7634

## References

[1] Bobulescu IA. Renal lipid metabolism and lipotoxicity. Curr Opin Nephrol Hypertens. (2010) 19:393–402. doi: 10.1097/MNH.0b013e32833aa4ac, 20489613 PMC3080272

[2] Escasany Elia Izquierdo-Lahuerta Adriana Medina-Gómez Gema (2018) Chapter 7 – kidney damage in obese subjects: oxidative stress and inflammation. London: Marti Moral Amelia del Aguilera García Concepción María Obesity Academic Press

[3] Kang HM Ahn SH Choi P Ko Y-A Han SH Chinga F . Defective fatty acid oxidation in renal tubular epithelial cells has a key role in kidney fibrosis development. Nat Med. (2014) 21:37. doi: 10.1038/nm.3762, 25419705 PMC4444078

[4] Mitrofanova A Merscher S Fornoni A. Kidney lipid dysmetabolism and lipid droplet accumulation in chronic kidney disease. Nat Rev Nephrol. (2023) 19:629–45. doi: 10.1038/s41581-023-00741-w, 37500941 PMC12926870

[5] Lucke VM. Renal disease in the domestic cat. J Pathol Bacteriol. (1968) 95:67–91. doi: 10.1002/path.1700950110, 5689371

[6] Modell W. Observations on the lipoids in the renal tubule of the cat. Anat Rec. (1933) 57:13–27. doi: 10.1002/ar.1090570104

[7] Foote JJ Grafflin AL. Quantitative measurements of the fat-laden and fat-free segments of the proximal tubule in the nephron of the cat and dog. Anat Rec. (1938) 72:169–79. doi: 10.1002/ar.1090720205

[8] Lobban MC. Some observations on the intracellular lipid in the kidney of the cat. J Anat. (1955) 89:92 14353800 PMC1244728

[9] Martino-Costa AL Malhão F Lopes C Dias-Pereira P. Renal interstitial lipid accumulation in cats with chronic kidney disease. J Comp Pathol. (2017) 157:75–9. doi: 10.1016/j.jcpa.2017.06.008, 28942307

[10] Hewer TF Harrison Matthews L Malkin T. (1949). Lipuria in tigers. In Proceedings of the Zoological Society of London, 924–928. Wiley Online Library.

[11] Newkirk KM Newman SJ White LA Rohrbach BW Ramsay EC. Renal lesions of nondomestic felids. Vet Pathol. (2011) 48:698–705. doi: 10.1177/0300985810382089, 20876911

[12] D'Arcy RL. (2018) Chronic Kidney Disease in Non-Domestic Felids in Australian Zoos [Doctoral dissertation, The University of Sydney]. Sydney eScholarship Repository.

[13] Macnider WD. Occurrence of stainable lipoid material in renal epithelium of animals falling in different age segments. Proc Soc Exp Biol Med. (1945) 58:326–8. doi: 10.3181/00379727-58-14941

[14] Quimby JM McLeland SM Cianciolo RE Lunn KF Lulich JP Erickson A . Frequency of histologic lesions in the kidneys of cats without kidney disease. J Feline Med Surg. (2022) 24:e472–80. doi: 10.1177/1098612X221123768 36475921 PMC10812332

[15] Bargmann W Krisch B Leonhardt H Mályusz M. Lipids in the proximal convoluted tubule of the cat kidney and the reabsorption of cholesterol. Cell Tissue Res. (1977) 177:523–38. doi: 10.1007/BF00220612, 189931

[16] Chapman KD Dyer JM Mullen RT. Biogenesis and functions of lipid droplets in plants: thematic review series: lipid droplet synthesis and metabolism: from yeast to man. J Lipid Res. (2012) 53:215–26. doi: 10.1194/jlr.R021436, 22045929 PMC3269164

[17] Maunsbach AB Wirsén C. Ultrastructural changes in kidney, myocardium and skeletal muscle of the dog during excessive mobilization of free fatty acids. J Ultrastruct Res. (1966) 16:35–54. doi: 10.1016/s0022-5320(66)80021-7, 5956756

[18] Olofsson S-O Boström P Andersson L Rutberg M Perman J Borén J. Lipid droplets as dynamic organelles connecting storage and efflux of lipids. Biochim Biophys Acta. (2009) 1791:448–58. doi: 10.1016/j.bbalip.2008.08.001, 18775796

[19] Bostrom P Andersson L Rutberg M Perman J Lidberg U Johansson BR . SNARE proteins mediate fusion between cytosolic lipid droplets and are implicated in insulin sensitivity. Nat Cell Biol. (2007) 9:1286–93. doi: 10.1038/ncb1648, 17922004

[20] Ducharme NA Bickel PE. Lipid droplets in lipogenesis and lipolysis. Endocrinology. (2008) 149:942–9. doi: 10.1210/en.2007-1713, 18202123

[21] Stannard SR Johnson NA. Insulin resistance and elevated triglyceride in muscle: more important for survival than 'thrifty' genes? J Physiol. (2004) 554:595–607. doi: 10.1113/jphysiol.2003.053926, 14608009 PMC1664785

[22] Else PL Hulbert AJ. An allometric comparison of the mitochondria of mammalian and reptilian tissues: the implications for the evolution of endothermy. J Comp Physiol B. (1985) 156:3–11. doi: 10.1007/bf00692920, 3836230

[23] Bartz R Li W-H Venables B Zehmer JK Roth MR Welti R . Lipidomics reveals that adiposomes store ether lipids and mediate phospholipid traffic. J Lipid Res. (2007) 48:837–47. 17210984 10.1194/jlr.M600413-JLR200

[24] Wölk M Fedorova M. The lipid droplet lipidome. FEBS Lett. (2024) 598:1215–25. doi: 10.1002/1873-3468.14874, 38604996

[25] Sakuma I Gaspar RC Nasiri AR Dufour S Kahn M Zheng J . Liver lipid droplet cholesterol content is a key determinant of metabolic dysfunction–associated steatohepatitis. Proc Natl Acad Sci USA. (2025) 122:e2502978122. doi: 10.1073/pnas.2502978122 40310463 PMC12067271

[26] Thannickal VJ Zhou Y Gaggar A Duncan SR. Fibrosis: ultimate and proximate causes. J Clin Invest. (2014) 124:4673–7. doi: 10.1172/JCI74368, 25365073 PMC4347226

[27] Wynn TA Ramalingam TR. Mechanisms of fibrosis: therapeutic translation for fibrotic disease. Nat Med. (2012) 18:1028–40. doi: 10.1038/nm.2807, 22772564 PMC3405917

[28] Lawson J Elliott J Wheeler-Jones C Syme H Jepson R. Renal fibrosis in feline chronic kidney disease: known mediators and mechanisms of injury. Vet J. (2015) 203:18–26. doi: 10.1016/j.tvjl.2014.10.009, 25475166

[29] Pan DA Hulbert AJ Storlien LH. Dietary fats, membrane phospholipids and obesity. J Nutr. (1994) 124:1555–65. doi: 10.1093/jn/124.9.1555, 8089723

[30] Stich V Berlan M. Physiological regulation of NEFA availability: lipolysis pathway. Proc Nutr Soc. (2004) 63:369–74. doi: 10.1079/pns2004350, 15294057

[31] Weinberg JM. Lipotoxicity. Kidney Int. (2006) 70:1560–6. doi: 10.1038/sj.ki.5001834, 16955100

[32] Whitby A Pabla P Shastri B Amugi L Del Río-Álvarez Á Kim DH . Characterisation of aberrant metabolic pathways in hepatoblastoma using liquid chromatography and tandem mass spectrometry (LC-MS/MS). Cancers. (2023) 15:5182. doi: 10.3390/cancers15215182, 37958356 PMC10648437

[33] Starr NJ Khan MH Edney MK Trindade GF Kern S Pirkl A . Elucidating the molecular landscape of the stratum corneum. Proc Natl Acad Sci USA. (2022) 119:e2114380119. doi: 10.1073/pnas.2114380119, 35298332 PMC8944899

[34] Kotowska AM Zhang J Carabelli A Watts J Aylott JW Gilmore IS . Toward comprehensive analysis of the 3D chemistry of *Pseudomonas aeruginosa* biofilms. Anal Chem. (2023) 95:18287–94. doi: 10.1021/acs.analchem.3c04443, 38044628 PMC10719885

[35] Hutchins PM Barkley RM Murphy RC. Separation of cellular nonpolar neutral lipids by normal-phase chromatography and analysis by electrospray ionization mass spectrometry. J Lipid Res. (2008) 49:804–13. doi: 10.1194/jlr.M700521-JLR200, 18223242 PMC2367097

[36] Magnusson CD Haraldsson GG. Ether lipids. Chem Phys Lipids. (2011) 164:315–40. doi: 10.1016/j.chemphyslip.2011.04.010, 21635876

[37] Yamazaki T Seyama Y Otsuka H Ogawa H Yamakawa T. Identification of alkyldiacylglycerols containing saturated methyl branched chains in the Harderian gland of guinea pig. J Biochem. (1981) 89:683–91. doi: 10.1093/oxfordjournals.jbchem.a133246, 7240136

[38] Fahy E Sud M Cotter D Subramaniam S. LIPID MAPS online tools for lipid research. Nucleic Acids Res. (2007) 35:W606–12. doi: 10.1093/nar/gkm324, 17584797 PMC1933166

[39] Mottram VH. Fatty infiltration of the cat's kidney. J Physiol. (1916) 50:380. doi: 10.1113/jphysiol.1916.sp001763, 16993351 PMC1420564

[40] Koopman R Schaart G Hesselink MK. Optimisation of oil red O staining permits combination with immunofluorescence and automated quantification of lipids. Histochem Cell Biol. (2001) 116:63–8. doi: 10.1007/s004180100297, 11479724

[41] Kruth HS. Localization of unesterified cholesterol in human atherosclerotic lesions. Demonstration of filipin-positive, oil-red-O-negative particles. Am J Pathol. (1984) 114:201 6198918 PMC1900338

[42] Schwarz T Shorten E Gennace M Saunders J Longo M Costa FS . CT features of feline lipiduria and renal cortical lipid deposition. J Feline Med Surg. (2021) 23:357–63. doi: 10.1177/1098612X20957161, 32960133 PMC8008399

[43] Rybin VG Imbs AB Demidkova DA Ermolenko EV. Identification of molecular species of monoalkyldiacylglycerol from the squid *Berryteuthis magister* using liquid chromatography–APCI high-resolution mass spectrometry. Chem Phys Lipids. (2017) 202:55–61. doi: 10.1016/j.chemphyslip.2016.11.008, 27894769

[44] Rico JE Samii SS Zang Y Deme P Haughey NJ Grilli E . Characterization of the plasma lipidome in dairy cattle transitioning from gestation to lactation: identifying novel biomarkers of metabolic impairment. Meta. (2021) 11:290. doi: 10.3390/metabo11050290, 33946522 PMC8147189

[45] Ma Z Onorato JM Chen L Nelson DW Yen C-LE Cheng D. Synthesis of neutral ether lipid monoalkyl-diacylglycerol by lipid acyltransferases[S]. J Lipid Res. (2017) 58:1091–9. doi: 10.1194/jlr.M073445, 28420705 PMC5454505

[46] Ermolenko E Latyshev N Sultanov R Kasyanov S. Technological approach of 1-O-alkyl-sn-glycerols separation from *Berryteuthis magister* squid liver oil. J Food Sci Technol. (2016) 53:1722–6. doi: 10.1007/s13197-015-2148-x, 27570298 PMC4984729

[47] Montague MJ Li G Gandolfi B Khan R Aken BL Searle SMJ . Comparative analysis of the domestic cat genome reveals genetic signatures underlying feline biology and domestication. Proc Natl Acad Sci USA. (2014) 111:17230–5. doi: 10.1073/pnas.1410083111, 25385592 PMC4260561

[48] Backus RC Thomas DG Fritsche KL. Comparison of inferred fractions of n-3 and n-6 polyunsaturated fatty acids in feral domestic cat diets with those in commercial feline extruded diets. Am J Vet Res. (2013) 74:589–97. doi: 10.2460/ajvr.74.4.589, 23531067

[49] Kennedy DG Kennedy S Blanchflower WJ Scott JM Weir DG Molloy AM . Cobalt-vitamin B12 deficiency causes accumulation of odd-numbered, branched-chain fatty acids in the tissues of sheep. Br J Nutr. (1994) 71:67–76. doi: 10.1079/bjn19940111, 7906141

[50] Izquierdo-Lahuerta A Martínez-García C Medina-Gómez G. Lipotoxicity as a trigger factor of renal disease. J Nephrol. (2016) 29:603–10. doi: 10.1007/s40620-016-0278-5, 26956132

